# Patterns of suicide mortality in England and Wales before and after the suicide of the actor Robin Williams

**DOI:** 10.1007/s00127-021-02059-z

**Published:** 2021-03-20

**Authors:** Alexandra Pitman, David S. Fink, Rob Whitley

**Affiliations:** 1 UCL Division of Psychiatry, Faculty of Brain Sciences, University College London, 6th Floor, Maple House, 149 Tottenham Court Road, London, W1T 7NF UK; 2 Camden and Islington NHS Foundation Trust, London, UK; 3 Department of Epidemiology, Mailman School of Public Health, Columbia University, New York, NY USA; 4 New York State Psychiatric Institute, New York, NY USA; 5 Department of Psychiatry, Douglas Mental Health University Institute, McGill University, Montreal, QC Canada

**Keywords:** Suicide, Broadcast media, Printed media, Celebrity, Media guidelines

## Abstract

**Purpose:**

There is international evidence supporting an association between sensational reporting of suicide and a subsequent increase in local suicide rates, particularly where reporting the death of a celebrity. We aimed to explore whether the observed increase in suicides in the United States, Canada and Australia in the 5 months following the 2014 suicide of the popular actor Robin Williams was also observed in England and Wales.

**Method:**

We used interrupted time-series analysis and a seasonal autoregressive integrated moving averages (SARIMA) model to estimate the expected number of suicides during the 5 months following Williams’ death using monthly suicide count data for England and Wales from the UK Office for National Statistics (ONS) 2013–2014.

**Results:**

Compared with the observed 2051 suicide deaths in all age groups from August to December 2014, we estimated that we would have expected 1949 suicides over the same period, representing no statistically significant excess.

**Conclusions:**

This finding is an outlier among previous studies and contrasts with the approximately 10% increase in suicides found in similar analyses conducted in other high-income English-speaking countries with established media reporting guidelines.

## Introduction

Media guidelines on the reporting of suicide [1] have arisen as a core feature of international suicide prevention strategies to address concerns about the increase in suicide rates observed after irresponsible media reporting of a suicide death [2, 3]. Evidence for this so-called Werther Effect [4] is strongest in relation to reporting the suicide of a celebrity [5, 6] and effects are thought to arise through a process of social transmission, particularly in young people [7, 8]. Such transmission effects include social identification with the deceased person, particularly where the same age or gender, or where the deceased was highly revered. It is also possible that widespread media reporting of suicide has the effect of normalising suicide as an accepted way of coping with adversity, and that the provision of information on suicide methods may increase the cognitive availability of a specific suicide method to a vulnerable individual.

A meta-analysis of 31 studies describing local changes in suicide rates before and after media reporting of non-fictional suicides found that the risk of suicide increased by 13% after extensive reporting [5]. In cases where the method used was reported, risk of suicide by the same method increased by 30% [5]. This meta-analysis, which included data from Australia, North America, Europe and Asia over the period 1947 to 2016, found that the magnitude of the elevated suicide risk did not vary by geographical region but did vary by time period, such that it was stronger after 2005 [5]. In cases of celebrity suicide, risk was of greater magnitude where the deceased was an entertainer rather than another type of well-known figure, such as an athlete or business leader [5]. However, risk did not appear to vary by whether they were famous within the country under study or internationally [5]. This evidence suggests that that national-level factors, whether relating to journalistic style, audience effects, or local suicide prevention resources, influence population suicide risk after media reporting of celebrity suicide.

To counter these effects, media guidelines [1] advise on preferred language and reporting styles, including avoidance of sensationalism and of details of the method, and the provision of information on sources of support. Such guidance was published by the World Health Organization (WHO) with the International Association for Suicide Prevention (IASP) in 2008 [1], updated in 2017 [9]. Individual countries have also developed their own media guidelines independently, including the United Kingdom (UK) [10, 11], the United States (US) [12, 13], Canada [14, 15] and Australia [16].

### Aims

Fears about the potential for wider transmission effects grew after the suicide of Robin Williams, an internationally popular US actor and comedian who died at the age of 63 years on 11 August 2014, following a history of neurological disease, depression, and substance misuse. His death was reported widely in the international media, prompting criticism of journalists in Britain [17–20] and elsewhere where they breached reporting guidelines by sensationalising the story and detailing the method used [21]. Time-series analyses of population data for the US [22], Canada [23], and Australia [24] described an approximately 10% increase in suicide deaths in the period after Robin Williams’ death. This was despite successful efforts in Canada [23] and Australia [24] to work with journalists to improve media coverage of suicide, yet only moderate adherence to reporting guidelines in the US [25]. To investigate consistency of findings, we aimed to explore whether similar patterns were observed in England and Wales and fill a gap in the literature.

## Method

We used monthly suicide mortality counts for England and Wales available from the UK’s Office for National Statistics (ONS) for 2014 [26] to report overall suicide trends in the 5 months following Robin Williams’ death (14 August 2014), a span of time used in previous similar analyses (August–December 2014) [22–24], which allows for the assessment of short- and medium-term impacts of the exposure. We also stratified by age group and by sex. These de-identified data are in the public domain.

We used interrupted time-series analysis to determine the expected number of suicides during August, September, October, November and December 2014, using a seasonal autoregressive integrated moving averages (SARIMA) model to account both for seasonal patterns and for autoregression, as consistent with other studies [22, 23]. To assess the difference in observed suicides and expected suicides over this 5-month period, we used a SARIMA (0,1,1) × (0,1,2)12 to model the log transformed observed number of suicides from January 2000 to July 2014 and forecasted the expected number of suicides, and 95% prediction intervals (CIs), from August 2014 to December 2014. All analyses were performed using R version 3.6.3 (Vienna, Austria).

## Results

**Fig. 1:**
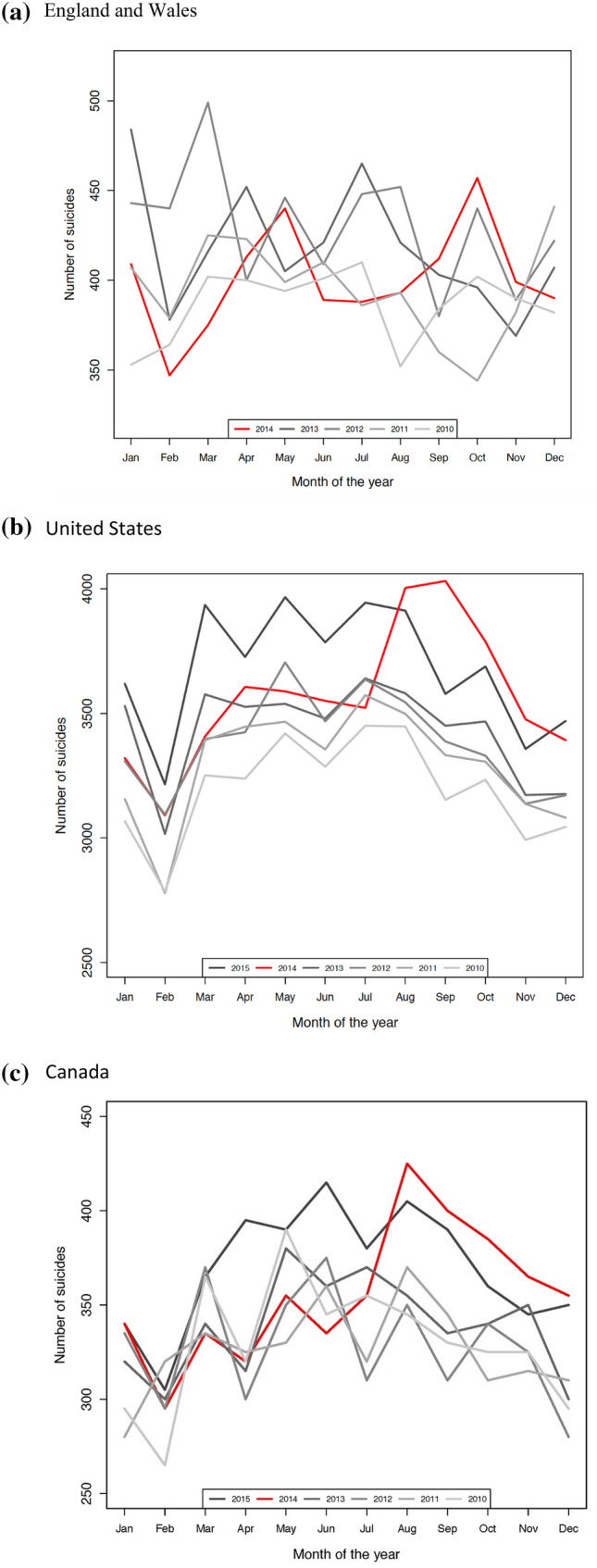
Absolute number of monthly suicide deaths over the period 2010 to 2014 in **a** England and Wales, **b** United States, and **c** Canada

On visual inspection of monthly plots of the absolute number of suicide deaths over the period 2010 to 2014 in England and Wales, no clear increase of suicides in August 2014 was observed when compared with August figures for earlier years or those for the United States and Canada (Fig. 1). Instead, a similar seasonal pattern was observed in each of the four years, with characteristic spring and summer peaks.

**Fig. 2:**
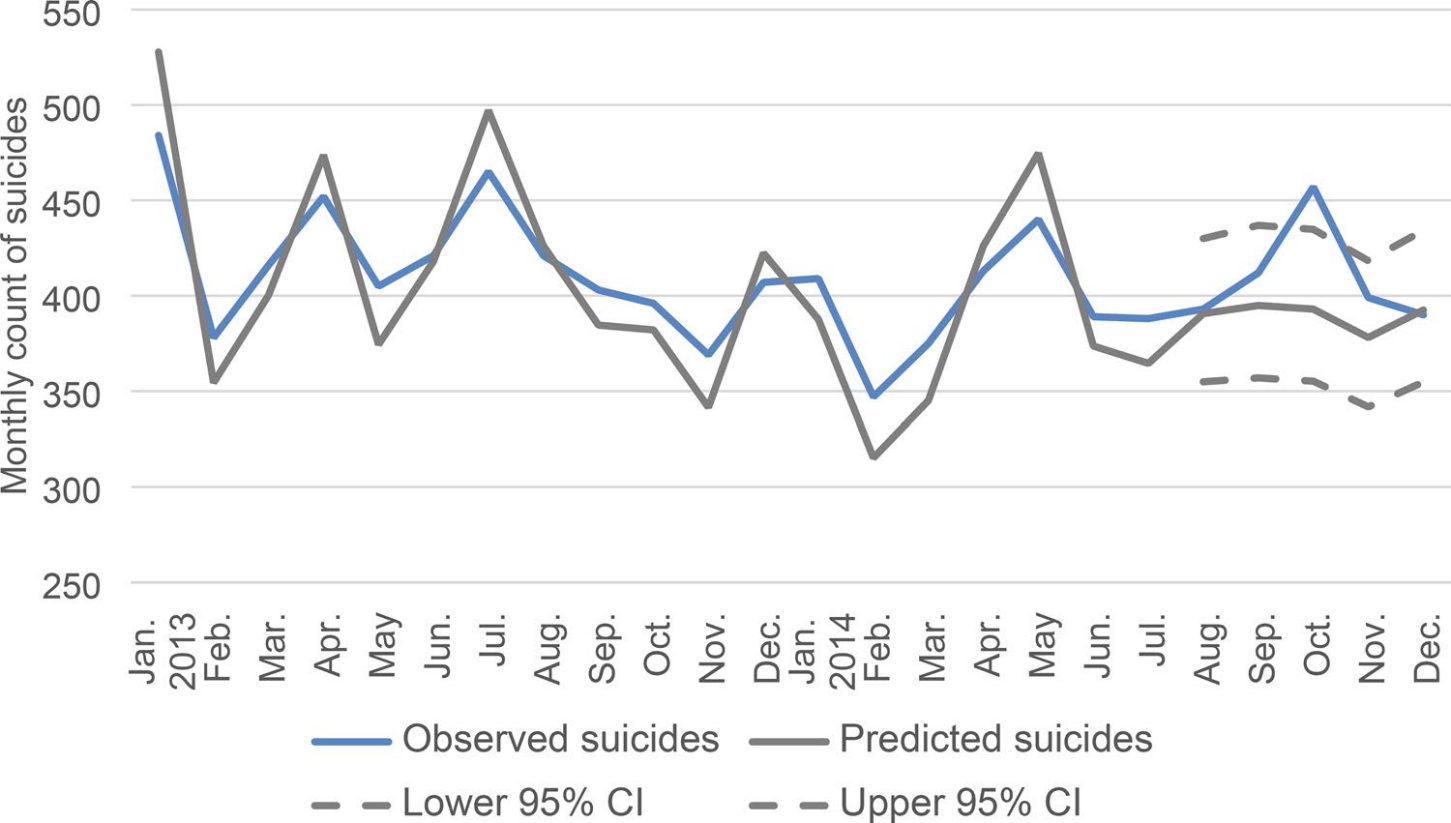
Observed and predicted monthly count of suicide deaths, England and Wales, 2013–2014

**Fig. 3:**
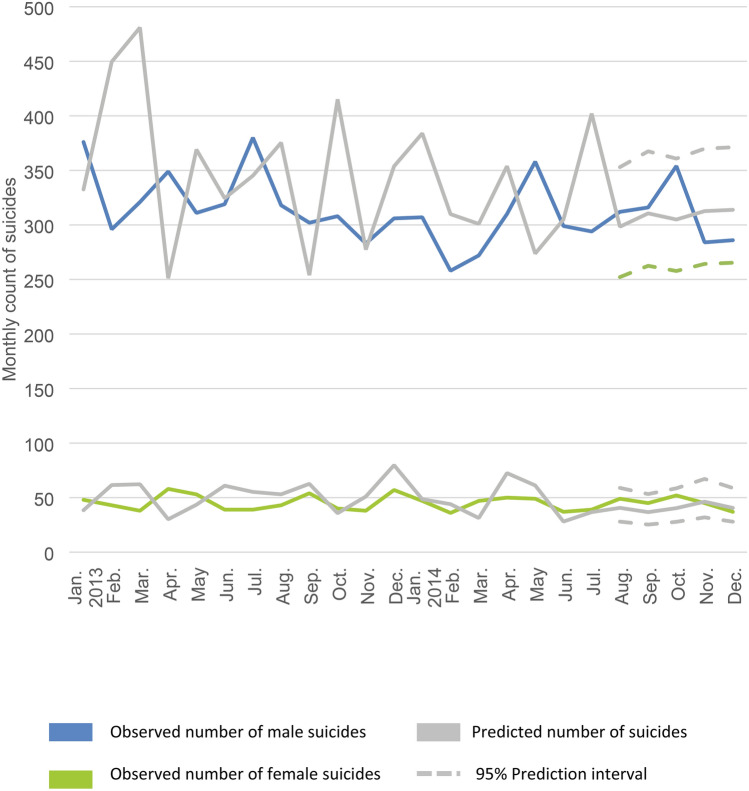
Observed and predicted monthly count of suicide deaths stratified by sex, England and Wales, 2013–2014

We estimated that we would expect 1949 suicides in all age groups in England and Wales from August to December 2014 (Table 1). When broken down by age group, observed suicides exceeded expected suicides for all age groups except adults aged 40–49 years (Table 1), but these differences were not significant. When broken down by month, observed suicides exceeded expected suicides for one month (October), in which the number of suicide deaths observed (*n* = 457) just exceeded the 95% prediction interval (355–435), but mirrored a similar October spike in the previous year. Over the whole 5-month period, we found no statistically significant difference between the observed and expected number of suicide deaths (Fig. 2), with the observed total of 2051 falling within the 95% prediction intervals for both men and women (Fig. 3) and across all age groups (Table 1). There was no evidence that residuals deviated from white noise behaviour (*Q* tests, *p* = 0.0001).

**Table 1:** Observed and expected number of suicides from August 2014 to December 2014 for males and females combined in England and Wales by age group

	Observed	Expected	Difference
Ages > 20 years			
August	12	10.11 (4.92, 20.76)	
September	20	10.14 (4.94, 20.82)	
October	9	9.35 (4.55, 19.21)	
November	16	10.54 (5.13, 21.65)	
December	12	12.92 (6.29, 26.53)	
Aug–Dec	**69**	**53.07**	15.93
Ages 20–29 years			
August	53	54.31 (38.03, 77.58)	
September	60	59.39 (41.58, 84.83)	
October	67	55.05 (38.54, 78.63)	
November	52	56.80 (39.76, 81.12)	
December	54	52.61 (36.83, 75.15)	
Aug–Dec	**286**	**278.16**	7.84
Ages 30–39 years			
August	62	63.52 (47.59, 84.77)	
September	75	61.64 (46.18, 82.26)	
October	73	64.75 (48.51, 86.41)	
November	70	73.05 (54.74, 97.50)	
December	63	69.28 (51.98, 92.32)	
Aug–Dec	**343**	**332.23**	10.77
Ages 40–49 years			
August	107	88.83 (68.39, 115.37)	
September	89	98.50 (75.81, 127.93)	
October	106	96.45 (74.27, 125.39)	
November	71	107.92 (83.09, 140.17)	
December	93	101.63 (78.24, 132.00)	
Aug–Dec	**466**	**493.33**	− 27.33
Ages 50–59 years			
August	68	70.73 (53.39, 93.69)	
September	79	82.07 (61.96, 108.72)	
October	89	78.30 (59.11, 103.72)	
November	83	74.34 (56.12, 98.47)	
December	81	78.92 (59.58, 104.54)	
Aug–Dec	**400**	**384.35**	15.65
Ages 60–69 years			
August	49	40.63 (28.01, 55.93)	
September	45	36.72 (25.31, 53.27)	
October	52	40.38 (27.83, 58.57)	
November	45	46.29 (31.91, 67.15)	
December	37	40.55 (27.96, 58.83)	
Aug–Dec	**228**	**204.57**	23.43
Ages 70–79 years			
August	19	24.11 (15.17, 38.30)	
September	23	24.85 (15.65, 39.48)	
October	39	23.80 (14.98, 37.82)	
November	31	24.83 (15.63, 39.45)	
December	30	27.30 (17.19, 43.37)	
Aug–Dec	**142**	**124.90**	17.10
Ages 80 and older			
August	23	20.50 (11.71, 35.89)	
September	21	20.65 (11.80, 36.14)	
October	22	20.20 (11.54, 35.36)	
November	31	19.95 (11.40, 34.93)	
December	20	19.73 (11.28, 34.52)	
Aug–Dec	**117**	**101.04**^**a**^	15.96
Total	2051	1971.65	

^**a**^Note that the total figure for expected number of suicides in this table differs from the 1949 quoted in the text because each age group is estimated separately, adding more uncertainty

## Discussion

### Main findings

This study, investigating suicide rates in England and Wales after the suicide of Robin Williams, found no evidence of an increase in suicides. This represents the first non-significant finding among time-series analyses for four high-income English-speaking countries addressing the same research question about the impact of media reporting of celebrity suicide on local suicide rates. In England and Wales the characteristic August peak in suicides was not significantly higher in 2014 than in previous years, indicating that reporting of the death of Robin Williams did not cause any discernible effect on suicide rates in Britain. There are a number of explanations for this finding, which contrasts with the increase in suicide mortality observed in the US, Australia and Canada. These explanations are informed by other literature, but are speculative given the absence of direct evidence surrounding the impact of Robin Williams’ suicide in Britain.

The first explanation is that Robin Williams was less popular in Britain than in the US, Canada or Australia, and that his death had less salience for British audiences or less prominence amongst other media content at that time. This would be consistent with the finding of a 2012 meta-analysis of studies exploring the effects of reporting celebrity suicides [6], in which the magnitude of risk was greater where the celebrity was of the same country, although this was not replicated in a later and more comprehensive 2020 meta-analysis [5]. A crude estimate of the comparative popularity of Robin Williams in the UK *versus* the US and Canada can be derived from a comparison of available box office figures for those countries [27], relating them to the national population denominator in the year of a film’s release [28]. Thus, Robin Williams’ 1993 film Mrs Doubtfire had a $0.84 taking per head of population in the US and Canada (taken together), compared with $0.55 per head of population in the UK. Although this suggests his lower prominence in the UK *versus* North America, obituaries and tributes in the British press described his enduring popularity in the UK since the 1970s [29], conveying a strong emotional valence to his death [30].

The second explanation is that reporting of his death in the UK media may have been more restrained than in the US, Canada or Australia, perhaps due to the proactive work of British media monitoring organisations such as Samaritans, resulting in better adherence of journalists to local guidelines [11, 31]. This argument is undermined by the strong criticism of British journalists by their peers at the time [17, 20], and the consistently high circulation in the UK of the more sensationalist tabloids such as The Sun and the Daily Mail [32]. A separate analysis of all media coverage of suicide in the UK and Ireland between 2012 and 2013 had also identified a tendency to over-report the suicides of celebrities [33]. Moreover, the rise in suicides in Australia and Canada after Robin Williams’ death appears to have been irrespective of objective improvements in suicide reporting in these countries over that period [23, 24]. We speculate that it is unlikely that UK audiences would have been significantly exposed to US reporting of Robin Williams’ suicide at that time, based on analyses of UK media consumption from that period**.** These describe the dominance and reach of the BBC in Britain via radio, television and online content, as well as the strong reach of local and national radio, television and newspapers [34]. Our finding of no increase in local suicide rates would only be considered as inconsistent with the findings of the related studies in the US [22, 25], Canada [23], and Australia [24] if we had evidence that the content of UK reporting of Robin Williams’ suicide matched that for those countries. However, studies evaluating the quality of British newspaper reporting of Robin Williams’ suicide over that period are lacking.

The third explanation is that the demographic group assumed to be most affected by the reporting of Robin Williams’s death, namely middle-aged men, may be better served by suicide prevention resources in the UK in times of mental health crisis by statutory services such as the National Health Service (NHS), and a range of voluntary sector organisations such as Samaritans, MIND, and the Campaign Against Living Miserably (CALM). Moreover, middle-aged men feature as a high-risk group in the English [35] and Welsh [36] suicide prevention strategies, with concerted efforts made to understand and address their needs [37]. Although this explanation is supported by the finding that 40–49 year olds were the only age group in this study to have recorded fewer suicide deaths than predicted, this hypothesis would require further testing, including comparative measurement of health service and voluntary sector utilisation and suicide prevention resources in the 5 months following Williams’ death.

A fifth explanation is the seasonal dip in newspaper circulation that occurs in the UK every August, at a time when many people are on holiday, and in the context of a longer-term fall in newspaper circulation figures [38]. However, the argument of lesser audience exposure is weakened by the observed switch from printed newspapers to the internet for news consumption in the UK over the period 2013 to 2016 [39]. A final explanation is that the Werther Effect does not operate in the British population in response to extensive media coverage of a celebrity’s suicide, but no other similar analysis has yet been conducted in the UK [5]. Our negative findings may be explained by the buffering effect of exogenous economic or social factors masking an underlying Werther Effect in the UK population over this period. It is also possible that misclassification of suicide deaths in the UK in this period, particularly given increasing use of narrative verdicts, may have obscured an increase in suicide deaths in the population [40].

### Strengths and limitations

The strengths of this study are that the analysis used whole population mortality data and a powerful method of time-series modelling to replicate prior studies on this important phenomenon, not previously investigated for any celebrities in the UK. The limitations include the issue of low specificity common to any other ecological study: that the individuals who died by suicide may not have been those exposed to the media content. Unlike similar studies [22, 23], our analysis did not stratify by suicide method due to resource constraints in accessing these data. Reporting of a non-fictional suicide method is associated with specific increases in suicides by the same method [5], and it would be helpful to investigate any changes in patterns of suicide method in the UK over the period of interest. We would, however, anticipate no significant changes in rates of suicide by hanging over this period, given the null findings of our main analysis and the high proportion of suicides by hanging (and by males) in the UK population at that time [26]. To have detected an increase in suicides by hanging, but not an increase across all suicide methods would suggest a decrease in other methods substantial enough to offset an increase in hangings, which does not seem plausible. As with other analyses, ours also relied on the assumption that a high proportion of the British population had been exposed to media messages about Robin Williams’ death. Finally, our study did not include a content analysis of UK media reports of Robin Williams’ suicide at that time.

### Clinical, policy and research implications

We cannot assume from this null finding that audiences in England or Wales are immune to the Werther Effect. More research is needed to explore the effect on suicide rates of media reporting of the suicide of a British celebrity, particularly in vulnerable populations such as those who self-harm. Beyond ecological studies, further work is required to understand individual-level effects on the audiences reached by media reports of a celebrity’s suicide in different settings. Recent irresponsible media reporting of the 2020 suicide of the British television presenter Caroline Flack [41] merits investigation of the effects of British media coverage on British suicide rates, particularly in women of the same age and younger. Heightened monitoring of the content and effects of social media coverage is also warranted, given concerns about this as a new and highly penetrative risk factor for suicide in youth audiences [42, 43]. In countries where clear evidence has been demonstrated for the Werther Effect [5], sustained efforts are needed to provide creative solutions for collaborating with journalists and social media users to support and maintain responsible reporting of suicide deaths. The 2020 edition of Samaritans media guidelines follows lengthy consultation with British journalists and industry leaders and includes a specific supplement on reporting celebrity suicides [10, 21]. Working closely with the media like this, the ambition of all those who work in suicide prevention is that journalists will take this opportunity to inform and educate the public about suicide, promoting help seeking and attitudinal changes in a way that mitigates the potential for social transmission of suicidal behaviour. This is particularly relevant during the coronavirus pandemic, in during which sensational coverage of suicidal behaviour could contribute to normalising this way of coping with pandemic-related difficulties [44].

## Conclusions

Our finding of no apparent increase in suicide deaths in the population of England and Wales after widespread reporting of the suicide of Robin Williams adds to the literature describing the relationship between celebrity suicide, the quality and quantity of media coverage of the event, and subsequent suicide deaths in the local population. Although the three other studies finding an excess of suicide deaths after Robin Williams’ death [22–24] cannot determine with certainty that these deaths were attributable to news media reporting, this body of literature does provide a rationale for working more closely with journalists in those countries to support responsible reporting of suicide in established and emerging media to positively influence vulnerable individuals. It also prompts further work in Britain to investigate the effects on local suicide rates of the reporting of the suicide of a British celebrity.

## Availability of data and material

ONS mortality statistics are publically available https://www.ons.gov.uk/peoplepopulationandcommunity/birthsdeathsandmarriages/deaths.

## Data Availability

R code available from the authors on request.
